# Incidence and outcome of acquired demyelinating syndromes in Dutch children: update of a nationwide and prospective study

**DOI:** 10.1007/s00415-018-8835-6

**Published:** 2018-03-22

**Authors:** C. L. de Mol, Y. Y. M. Wong, E. D. van Pelt, I. A. Ketelslegers, D. P. Bakker, M. Boon, K. P. J. Braun, K. G. J. van Dijk, M. J. Eikelenboom, M. Engelen, K. Geleijns, C. A. Haaxma, J. M. F. Niermeijer, E. H. Niks, E. A. J. Peeters, C. M. P. C. D. Peeters-Scholte, B. T. Poll-The, R. P. Portier, J. F. de Rijk-van Andel, J. P. A. Samijn, H. M. Schippers, I. N. Snoeck, H. Stroink, R. J. Vermeulen, A. Verrips, F. Visscher, J. S. H. Vles, M. A. A. P. Willemsen, C. E. Catsman-Berrevoets, R. Q. Hintzen, R. F. Neuteboom

**Affiliations:** 1 Department of Neurology, MS Centre ErasMS, Erasmus MC, Rotterdam, The Netherlands; 2 Department of Paediatric Neurology, VU Medical Centre, Amsterdam, The Netherlands; 3 Department of Paediatric Neurology, UMCG, Groningen, The Netherlands; 4 Department of Paediatric Neurology, University Medical Centre Utrecht, Utrecht, The Netherlands; 5 Department of Paediatrics, Rijnstate Hospital, Arnhem, The Netherlands; 6 Department of Neurology, Westfriesgasthuis, Hoorn, The Netherlands; 7 Department of Paediatric Neurology, Academic Medical Centre Amsterdam, Amsterdam, The Netherlands; 8 Department of Paediatric Neurology, Radboud UMC, Nijmegen, The Netherlands; 9 Department of Neurology, Elisabeth-Tweesteden Hospital, Tilburg, The Netherlands; 10 Department of Neurology, Leiden University Medical Centre, Leiden, The Netherlands; 11 Department of Paediatric Neurology, Juliana Children Hospital/Haga Hospital, The Hague, The Netherlands; 12 Department of Neurology, Medical Spectrum Twente, Enschede, The Netherlands; 13 Department of Neurology, Amphia Hospital, Breda, The Netherlands; 14 Department of Neurology, Maasstad Hospital, Rotterdam, The Netherlands; 15 Department of Neurology, St. Antonius Hospital, Nieuwegein, The Netherlands; 16 Department of Neurology, Canisius-Wilhelmina Hospital, Nijmegen, The Netherlands; 17 Department of Neurology, Maastricht UMC, Maastricht, The Netherlands; 18 Department of Paediatric Neurology, Admiraal de Ruyter Hospital, Goes, The Netherlands; 19 Paediatric Neurology, Erasmus MC-Sophia, P.O. Box 2060, 3000 CB Rotterdam, The Netherlands

**Keywords:** Children, Multiple sclerosis, Acquired demyelinating syndromes, Epidemiology, Outcome

## Abstract

**Introduction:**

Acquired demyelinating syndromes (ADS) are immune-mediated demyelinating disorders of the central nervous system in children. A nationwide, multicentre and prospective cohort study was initiated in the Netherlands in 2006, with a reported ADS incidence of 0.66/100,000 per year and MS incidence of 0.15/100,000 per year in the period between 2007 and 2010. In this study, we provide an update on the incidence and the long-term follow-up of ADS in the Netherlands.

**Methods:**

Children < 18 years with a first attack of demyelination were included consecutively from January 2006 to December 2016. Diagnoses were based on the International Paediatric MS study group consensus criteria. Outcome data were collected by neurological and neuropsychological assessments, and telephone call assessments.

**Results:**

Between 2011 and 2016, 55/165 of the ADS patients were diagnosed with MS (33%). This resulted in an increased ADS and MS incidence of 0.80/100,000 per year and 0.26/100,000 per year, respectively. Since 2006 a total of 243 ADS patients have been included. During follow-up (median 55 months, IQR 28–84), 137 patients were diagnosed with monophasic disease (56%), 89 with MS (37%) and 17 with multiphasic disease other than MS (7%). At least one form of residual deficit including cognitive impairment was observed in 69% of all ADS patients, even in monophasic ADS. An Expanded Disability Status Scale score of ≥ 5.5 was reached in 3/89 MS patients (3%).

**Conclusion:**

The reported incidence of ADS in Dutch children has increased since 2010. Residual deficits are common in this group, even in monophasic patients. Therefore, long-term follow-up in ADS patients is warranted.

## Introduction

Acquired demyelinating syndromes (ADS) are immune-mediated demyelinating disorders of the central nervous system (CNS) in children [1, 2]. ADS encompass a wide spectrum of neurological symptoms depending on the location of inflammation and the severity of demyelination. As the clinical symptoms overlap in this spectrum, international consensus criteria have been proposed in 2007 to aid in diagnosis and distinction between subtypes [3]. These criteria were revised in 2012 [4]. In addition, new findings in the past few years added valuable insights into paediatric ADS and its subtypes, including the identification of new biomarkers such as anti-myelin oligodendrocyte glycoprotein antibodies (MOG-ab) [2, 5] and the identification of new clinical subtypes as acute disseminated encephalomyelitis followed by optic neuritis (ADEM-ON) [6].

ADS may remain monophasic after the first event. Yet, 15–32% of these children will fulfil the diagnostic criteria for paediatric MS within 5 years after the initial attack [1, 2, 7, 8]. Multiple aspects of outcome of paediatric MS patients have been described before, including the rate of disease progression in Expanded Disability Status Scale (EDSS) scores [9, 10], cognitive performance [11–13], decreased motor performance [14, 15], and neuropsychiatric complaints like fatigue and mood disorders [14, 16]. However, studies describing the long-term outcome of other ADS subtypes are scarce.

In the Netherlands, a multicentre and prospective study was established in 2006 with national coverage for children with a first demyelinating event. Incidence estimates of paediatric ADS and multiple sclerosis have been reported in our prior work for the period between 2007 and 2010 [17]. However, the number of patients who will be diagnosed with MS will likely increase with longer follow-up time. Furthermore, an increasing MS incidence in children has been reported in specific regions [18, 19]. Therefore, we aim to re-assess the incidence and presenting characteristics of ADS and its subtypes in the Netherlands. Second, we aim to provide long-term follow-up data of the patients included in our prospective and multicentre cohort in the Netherlands.

## Methods

### Patient inclusion

Children younger than 18 years, residing in the Netherlands, and experiencing a first inflammatory demyelinating event of the CNS in the period from 2006 to 2016 have been included in this study. All patients are participants of the PROUD-kids study (PRedicting the OUtcome of a Demyelinating event in children), a prospective, multicentre and observational cohort study. Paediatric neurologists of the eight Dutch academic hospitals and of ten non-academic hospitals took part in this study and included patients to reach nationwide coverage.

Diagnoses were made using the revised criteria proposed by the International Pediatric Multiple Sclerosis Study Group (IPMSSG) [4]. Patients with alternative diagnoses were excluded (e.g. systemic autoimmune diseases, infectious diseases or metabolic diseases). Patients were classified as neuromyelitis optica spectrum disorder (NMOSD) as presenting phenotype if either patients were tested seropositive for anti-aquaporin 4 antibodies (AQP4-ab), or when AQP4-ab-negative patients presented with simultaneous optic neuritis (either unilateral or bilateral) and transverse myelitis (TM) with at least three segments.

### Baseline parameters

At inclusion, demographic and clinical information of each patient was gathered. Demographic characteristics consisted for example of ethnic background, date and place of birth and family history on familial autoimmune diseases. Clinical characteristics consisted of presenting symptoms, reported infection or vaccination in the preceding 4 weeks, acute treatment and hospitalization. MRI images, serum and CSF parameters were also reviewed when available for diagnostics or evaluation.

### Follow-up parameters

If patients were not referred to the paediatric MS centre for follow-up, the follow-up data of the patients were provided by the treating physician (e.g. clinical letters) and by interviewing the parents through telephone every 2 years after disease onset.

Cognitive impairment (CI) and residual neurological deficits were assessed using the most recent neuropsychological assessment (NPA) performed by a paediatric clinical neuropsychologist, and neurological examination by a paediatric neurologist. NPAs were being performed appropriately for age. During the NPA, at least six of the following cognitive domains were being assessed for the presence of cognitive deficits: behaviour, language, intelligence, attention and concentration, memory, executive control functions and visuospatial abilities. Children were classified as cognitive impaired if at least one of these domains was affected.

If data on neurological examination or NPA were not available, a standardized questionnaire was administered asking parents or patients about the presence of sensory complaints, motor deficits (e.g. complaints regarding paresis, ataxia, balance problems), bladder complaints (e.g. urge incontinence), maximum walking distance and cognitive impairment (including negatively affected school performance).

The Expanded Disability Status Scale is widely used to express disability of patients with MS diagnosis [20]. EDSS 5.5 stands for a walking distance of maximum 100 m, without aid or rest.

### Antibody testing

Serum AQP4-ab and MOG-ab were tested with cell-based assays (CBA) provided in a central laboratory as described previously [21, 22]. Patients were tested for regular diagnostics, or retrospectively when serum of the patient was still available.

### Ethics approval

This study was approved by the Medical Ethical Committees of the Erasmus MC in Rotterdam and the other participating centres. Written informed consent was obtained from parents and also from patients if aged > 12 years at presentation.

### Statistical analysis

Demographic data of the general Dutch population were provided by Statistics Netherlands [23]. These data were used to calculate the incidence of ADS and its subtypes in the period of 2011–2016 in the Netherlands. Statistical analyses were performed using IBM SPSS 21. Figures were made using Graphpad Prism 5.

Chi-square and when appropriate Kruskal–Wallis tests were used to test differences in demographic and clinical characteristics between the different subtypes. For differences in numerical data between subtypes the ANOVA test was used, and when necessary the Mann–Whitney *U* test. To compare the ethnic background of the patients with the Dutch population we used a *Z* test, with data provided by Statistics Netherlands [23]. Results were considered significant if *p* < 0.05. Missing data were removed from the analyses in all subgroups.

## Results

### Incidence

**Fig. 1:**
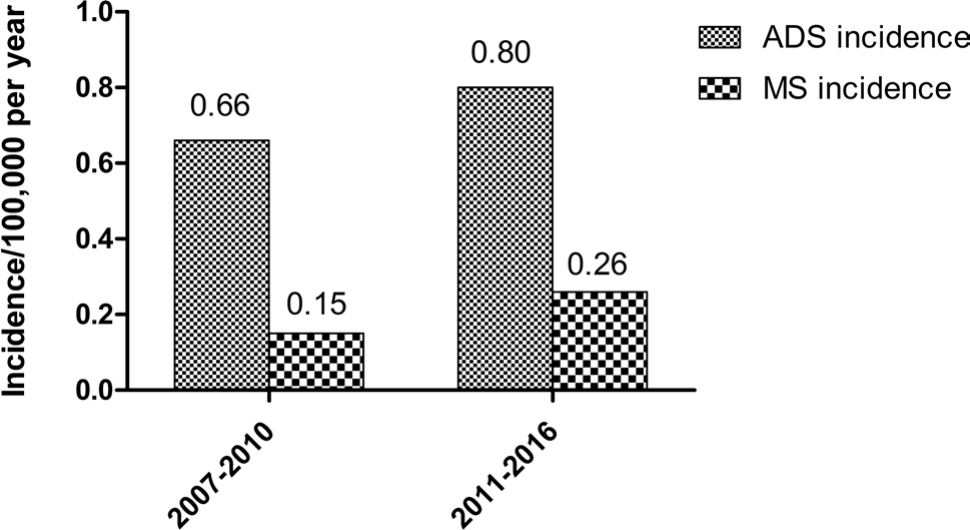
Incidence of acquired demyelinating syndromes (ADS) and multiple sclerosis (MS) in the Netherlands in 2007–2010 and 2011–2016

From January 1, 2011 to December 31, 2016, 165 ADS patients were reported of which 55 (33%) received an MS diagnosis during FU. In this period, the incidence of ADS was 0.80/100,000 per year, ADEM incidence was 0.23/100,000 per year and MS incidence was 0.26/100,000 per year. An overview of the calculated incidences is shown in Fig. 1.

### First presentation of ADS

**Fig. 2:**
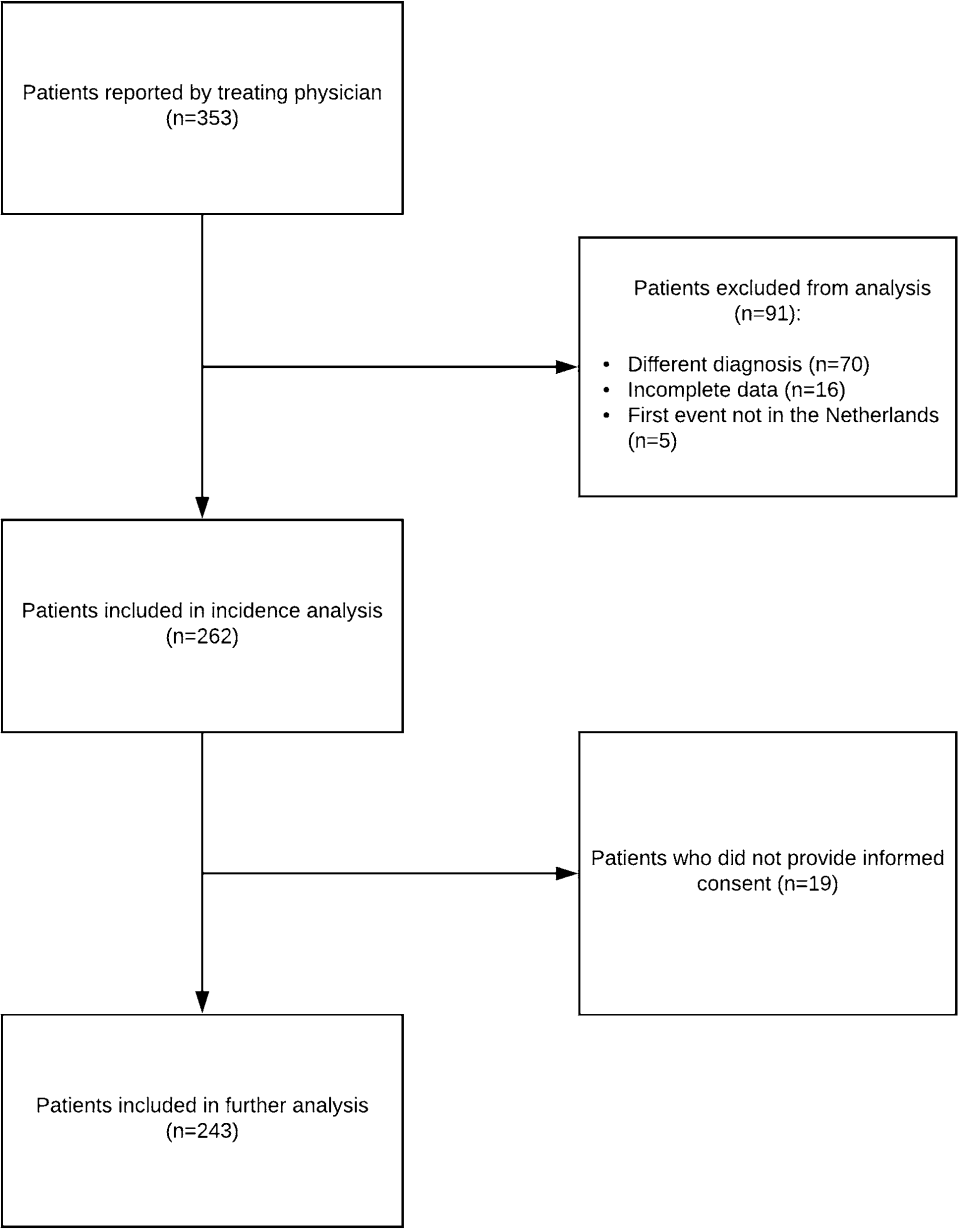
Flowchart describing the selection process

From January 1, 2006 to December 31, 2016, 353 patients were eligible. Of these patients, 243 patients with a first demyelinating event were included in the study (Fig. 2). Presenting phenotypes consisted of optic neuritis (ON; *n* = 55, 23%; from which 16/55 bilateral ON, 29%), transverse myelitis (TM; *n* = 23, 9%); other monofocal clinically isolated syndromes (CIS; *n* = 37, 15%), polyfocal CIS (*n* = 47, 19%), acute disseminated encephalomyelitis (ADEM; *n* = 70, 29%) and NMOSD (*n* = 11, 5%).

Regarding the age of onset, children with ADEM were significantly younger than the other presenting phenotypes (*p* < 0.001) and presented more often after a reported preceding infection (*p* < 0.001). The latter also applied to children who experienced a TM as first event (*p* = 0.01).

The ratio between females and males in all ADS patients did not differ significantly between the presenting phenotypes. When the ADS patients are divided into a group aged < 11 years (*n* = 104) and a group > 11 years (*n* = 139), the female:male ratio differed significantly (1.02:1 versus 1.76:1, *p* = 0.04).

Seventy-eight patients (32%) were of non-Caucasian origin. This proportion was significantly higher than the proportion of children of non-Caucasian origin (17%) in the general paediatric population in the Netherlands (*Z* = 5.1, *p* < 0.001) [23]. Most of the non-Caucasian patients were of African (29%) or Middle-eastern (23%) ethnicity.

Forty-eight percent of all patients had a positive familial history of autoimmune diseases (first- and second-grade family members). Forty-eight (20%) patients reported the presence of rheumatoid arthritis in their family, 40 (16%) reported thyroid diseases, 35 (14%) the presence of other autoimmune diseases (e.g. Wegener’s disease and Crohn’s disease), 15 (6%) the presence of MS, 14 (6%) diabetes mellitus type 1 and 4 (2%) the presence of optic neuritis (ON). No significant difference was observed between the presenting phenotypes considering the familial history (*p* = 0.3).

In all ADS patients, disease onset in winter was most prevalent (32%), compared to spring (28%), summer (22%) and autumn (17%). MOG antibodies were found to be positive in 31 of the 146 tested patients (21%). When comparing the presenting phenotypes, MOG antibodies were most frequently found in patients who presented with ADEM and NMOSD (*p* < 0.001).

Detailed patient characteristics are displayed in Table 1.

**Table 1:** Presenting phenotypes and demographic characteristics

	ON (*n* = 55)	TM (*n* = 23)	CIS monofocal (*n* = 37)	Polyfocal CIS (*n* = 47)	ADEM (*n* = 70)	NMOSD (*n* = 11)	*p* value*
Female, *n* (%)	30/55 (55)	15/23 (65)	23/37 (62)	29/47 (62)	35/70 (50)	7/11 (64)	0.7
Age at onset, years, median (IQR)	13.0 (9.6–15.8)	12.7 (4.5–16.1)	14.9 (12.0–16.2)	14.3 (9.4–15.9)	4.2 (2.6–6.1)	12.1 (9.7–16.3)	< 0.001^a^
Reported infection < 4 weeks prior to first event, *n* (%)	11/52 (21)	11/22 (50)	6/34 (18)	14/45 (31)	40/69 (58)	2/11 (18)	< 0.001^a^
							0.01^b^
Reported vaccination < 4 weeks prior to first event, *n* (%)	1/53 (2)	1/22 (5)	1/36 (3)	1/43 (1)	3/69 (4)	0/11 (0)	0.9
Presence of familial autoimmune diseases, *n* (%)	26/54 (48)	9/22 (41)	21/34 (62)	18/47 (38)	33/69 (48)	7/11 (64)	0.3
Use of acute immunomodulatory treatment, *n* (%)	37/55 (67)	19/23 (83)	15/36 (42)	30/44 (68)	61/70 (87)	11/11 (100)	< 0.001
Average amount of days in the hospital, median (IQR)	3.0 (0.0–5.0)	11.0 (5.0–22.0)	3.0 (0.0–6.5)	5.0 (0.0–10.5)	12.0 (6.8–21.0)	23.0 (5.0–23.0)	< 0.001
Total MS cases, *n* (%)	23/55 (42)	5/23 (22)	30/37 (81)	30/47 (64)	1/70 (1)	0/11 (0)	< 0.001
Relapsing disease, *n* (%)	23/55 (42)	4/23 (17)	25/37 (68)	27/47 (57)	8/70 (11)	3/11 (27)	< 0.001
Presence of MOG antibodies, *n* (%)	4/31 (13)	1/15 (7)	1/20 (5)	3/34 (9)	17/39 (44)	5/7 (71)	< 0.001
Presence of AQP4 antibodies, *n* (%)	0/37 (0)	0/17 (0)	0/12 (0)	0/23 (0)	0/36 (0)	5/11 (46)	< 0.001

*ON* optic neuritis, *TM* transverse myelitis, *ADEM* acute disseminated encephalomyelitis, *NMOSD* neuromyelitis optica spectrum disorder, *CIS* clinically isolated syndrome, *MS* multiple sclerosis, *MOG* anti-myelin oligodendrocyte glycoproteins, *AQP4* anti-aquaporin 4, *IQR* interquartile range, *n* number

*p value < 0.05 is considered statistically significant

^a^ADEM compared to the other presenting phenotypes

^b^TM compared to the other presenting phenotypes (excluding ADEM)

### Follow-up

For the follow-up analysis, we divided all patients into the following categories: monophasic disease, MS and multiphasic non-MS disease (Table 2). The median follow-up time of all patients was 55 months (IQR 28–84).

**Table 2:** Follow-up characteristics of the ADS patients

	Monophasic disease (*n* = 137)	MS (*n* = 89)	Multiphasic non-MS (*n* = 17)	*p* value*
Amount of relapses, median (IQR)	n/a	2.0 (1.0–3.5)	3.0 (1.5–4.0)	0.12
Length of follow-up in months, median (IQR)	47 (22–81)	61 (38–90)	71 (32–102)	0.01
Ethnicity,* n* (%)				< 0.001^a^
European	106 (77)	44 (49)	14 (82)	
Middle-eastern	7 (5)	11 (12)	0 (0)	
African	5 (4)	19 (21)	0 (0)	
South-American	1 (1)	1 (1)	2 (12)	
Caribbean	1 (1)	3 (3)	0 (0)	
Asian	3 (2)	2 (2)	0 (0)	
Mixed	13 (10)	9 (10)	1 (6)	
Unknown	1 (1)	0 (0)	0 (0)	
Use of immunomodulatory treatment > 1 year,* n* (%)	7/137 (5)	73/89 (82)	12/17 (71)	< 0.001^a^
Use of second-line immunomodulatory treatment,* n* (%)	1/137 (1)	28/89 (32)	5/17 (29)	< 0.001^a^
Presence of anti-MOG antibodies,* n* (%)	24/82 (29)	0/55 (0)	7/9 (78)	< 0.001^a^
Presence of anti-AQP4 antibodies,* n* (%)	3/83 (4)	0/37 (0)	2/16 (13)	0.09^a^

*MS* multiple sclerosis, *IQR* interquartile range, *MOG* anti-myelin oligodendrocyte glycoprotein, *AQP4* anti-aquaporin 4, *n* number, *EDSS* Expanded Disability Status Scale of 5.5 stands for a walking distance of about 100 m, without aid or rest

*p value < 0.05 is considered statistically significant

^a^Comparison between monophasic patients and MS

#### Monophasic patients

One hundred and thirty-seven patients remained monophasic (137/243, 56%), including ADEM (*n* = 62, 45%) ON (*n* = 27, 20%; from which 11/27 bilateral ON, 41%), TM (*n* = 17, 12%), CIS (*n* = 7, 5%), polyfocal CIS (*n* = 16, 12%), and monophasic NMOSD (*n* = 8, 6%). Of these NMOSD patients, three were tested seropositive for AQP4-ab and four were seropositive for MOG-ab. Seven monophasic patients received chronic immunosuppressive therapy: this was initiated in all AQP4-ab-positive patients, and in one of the MOG-ab-positive patients due to the disease severity at onset (Table 2). Two CIS patients received disease-modifying treatment (DMT) because of suspected risk of future MS. One patient had a LETM that required ICU admission and ventilation, and was, therefore, given chronic immunosuppressive therapy for 1 year.

#### MS patients

Eighty-nine patients were diagnosed with MS in our cohort (37%), of which 87 received the diagnosis within 5 years of follow-up. Of the 89 MS patients, 70 individuals developed a second attack during follow-up, and were thus diagnosed with clinically definite MS (CDMS). In these patients, the median time to CDMS was 9 months (IQR 4–27). After 2 years of follow-up, 74 percent of the MS patients developed CDMS. No patients with MS had a primary progressive disease course. Only one MS patient had an ADEM as first presentation. After dividing the MS patients into two groups, aged over or below 11 years, the sex ratio showed a trend towards significance (*p* = 0.07), with more girls in the older MS group.

Compared to monophasic ADS, patients who received MS diagnosis during follow-up were more often of non-Caucasian origin (*p* < 0.001) (Table 2). The calculated MS incidence for patients of non-Caucasian origin was 0.78/100,000 per year in the period from 2011 to 2016, compared to 0.16/100,000 per year in children of Caucasian origin.

At last follow-up, 73/89 MS patients were on DMT for the duration of at least 1 year and in 28 patients second-line treatment was started (Table 2). Of these 28 patients, 22 started using second-line treatment because of high MS activity, either on MRI or clinically, five because of side effects of first-line treatment and one due to the participation in an international paediatric MS drug trial.

#### Multiphasic non-MS patients

**Fig. 3:**
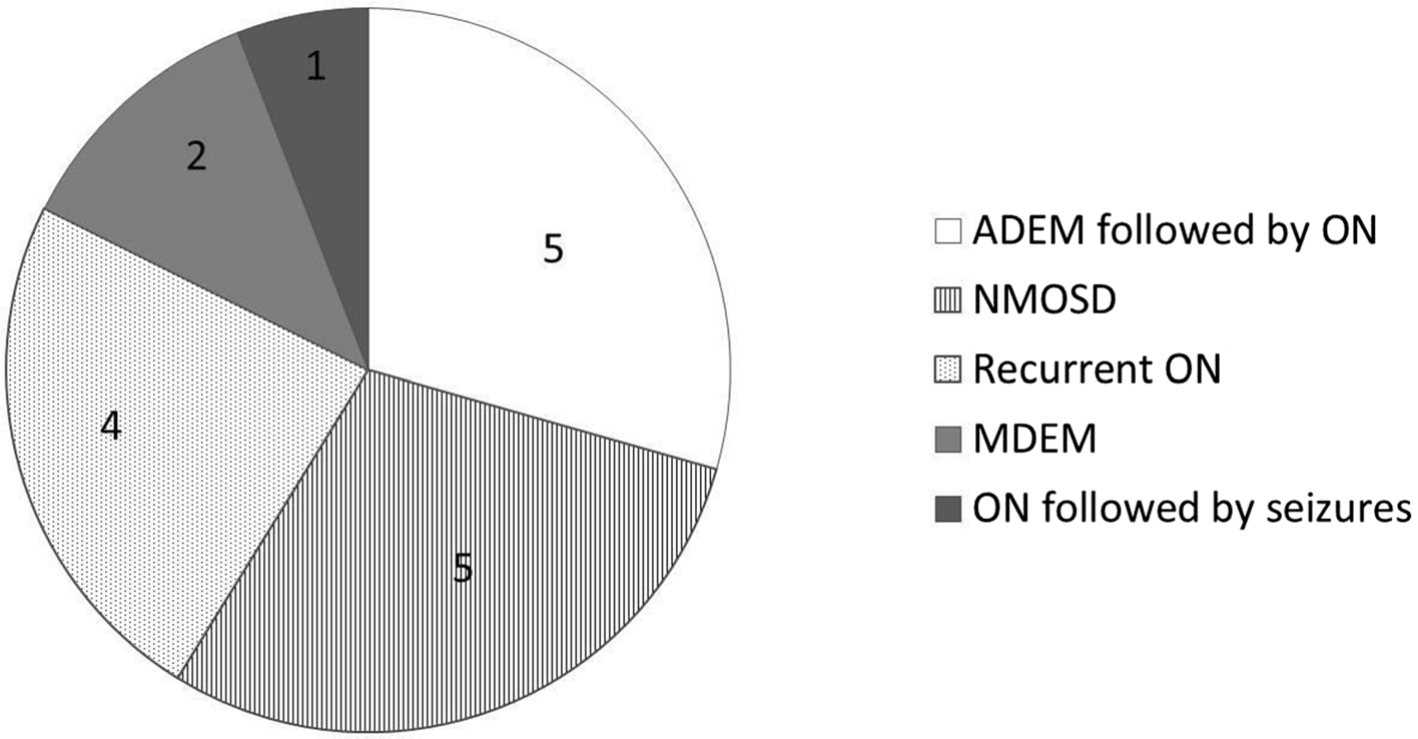
Distribution of clinical subtypes of patients diagnosed with multiphasic non-MS (*n* = 17). *ADEM* acute disseminated encephalomyelitis, *ON* optic neuritis, *NMOSD* neuromyelitis optica spectrum disorder, *MDEM* multiphasic acute disseminated encephalomyelitis

The patients with multiphasic non-MS disease consisted of five patients who were diagnosed with ADEM-ON, five with NMOSD (AQP4 *n* = 2, MOG *n* = 1), four with recurrent ON, two with multiphasic disseminated encephalomyelitis (MDEM) and one with ON followed by seizures (once secondary generalized convulsion, once focal epilepsy) (Fig. 3). Of these 17 patients, 12 used chronic immunomodulatory treatment > 1 year. Seven remained on first-line treatment (e.g. azathioprine, mycophenolate) and five patients were switched to second-line treatment (e.g. rituximab *n* = 3, monthly intravenous immunoglobulins (IVIG) *n* = 2).

#### Residual neurological deficits

Overall, physicians or parents reported at least one form of residual deficit at the last follow-up in 162/235 (69%) ADS patients, including 71/86 (83%) of the MS patients, 76/133 (57%) of the monophasic patients and 15/16 (94%) of the multiphasic non-MS patients. Residual neurological deficits were significantly more observed in MS patients compared to monophasic patients (*p* < 0.001).

In the mono-ADS group, residual deficits were most often present in patients with TM and NMOSD (*p* = 0.02). From the monophasic patients with a TM and residual deficits 11/14 had suffered from a longitudinal extended transverse myelitis. In MS and multiphasic non-MS patients, no significant difference was found in residual deficits between the presenting phenotypes.

**Fig. 4:**
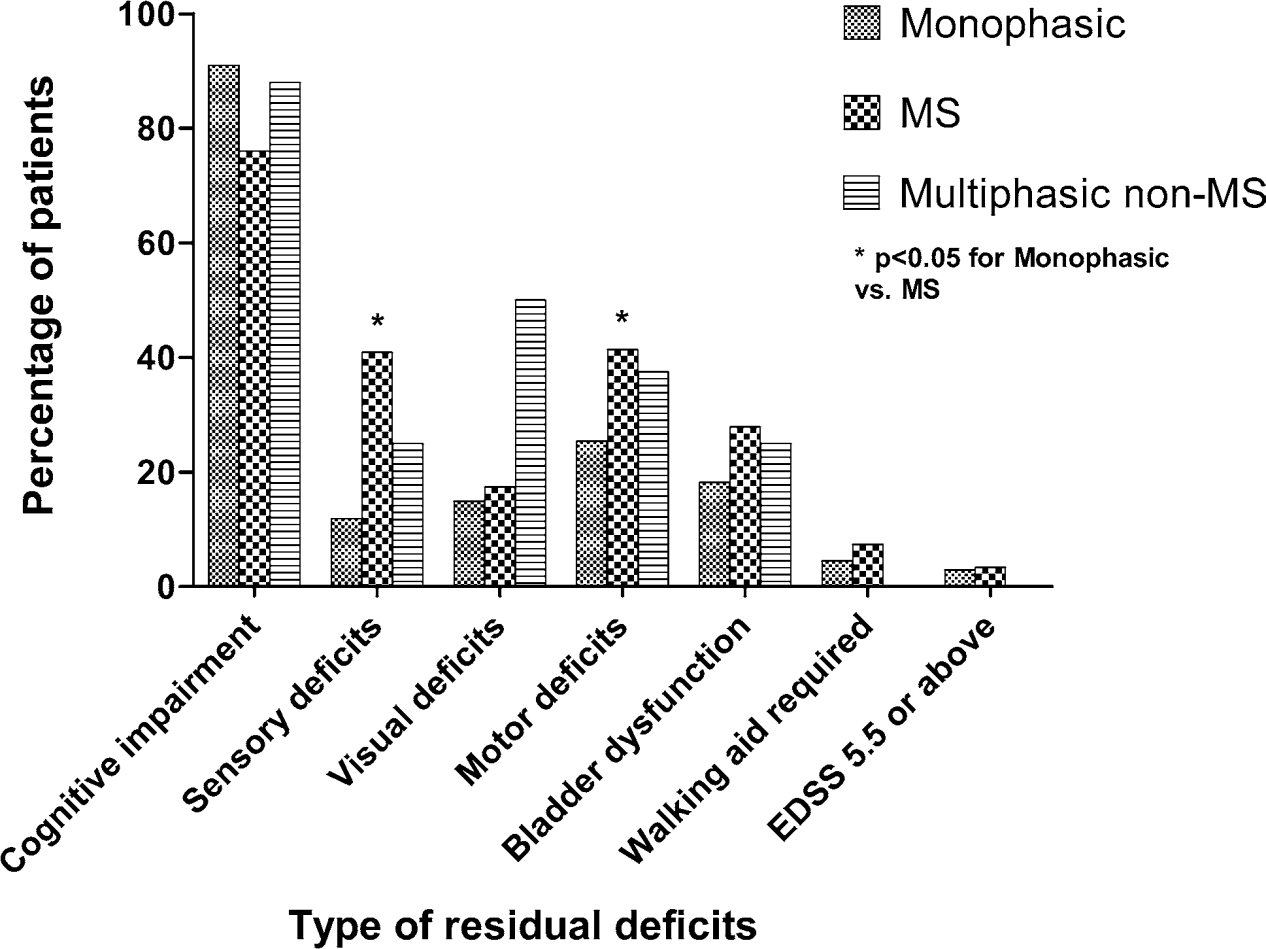
Residual deficits of ADS patients at last follow-up. Definition: patients were classified as cognitive impaired if they had a deficit in at least one cognitive domain, tested by a neuropsychological assessment

Specific differences between the three categories were observed: MS patients reported significantly more sensory deficits and motor deficits compared to monophasic patients (*p* < 0.05) (Fig. 4). Yet in multiphasic non-MS patients visual deficits and cognitive impairment were mostly reported at last follow-up.

#### Cognitive deficits

Thirty-two percent of all included patients (78/243) underwent an NPA (32/137 mono-ADS, 38/89 MS and 8/17 multiphasic non-MS).

At least one of the cognitive domains was affected in 65 of the tested patients (83%), respectively, in 29/32 (91%) monophasic ADS, 29/38 (76%) MS and 7/8 (88%) multiphasic non-MS. Three or more cognitive domains were affected in 18/32 (56%) monophasic patients, 22/38 (58%) MS patients and in 6/8 (75%) multiphasic non-MS patients. The three most commonly affected domains in monophasic patients were intelligence, attention and concentration and memory, in MS patients language, attention and concentration and memory and in multiphasic non-MS patients attention and concentration, memory and executive control functions. Median time till NPA from disease onset was 15 months (IQR 6–32). In monophasic patients the median time was 25 months (IQR 11–62), in MS patients 11 (IQR 6–24) and in multiphasic non-MS patients 10 (IQR 3–22).

Data on school performance were available in 216/243 patients (monophasic patients: *n* = 124, MS: *n* = 76, multiphasic non-MS patients: *n* = 16). Negatively affected school performance was reported by 63/216 (29%) of the participants: 37/124 (30%) of the monophasic patients, 16/76 (21%) of the MS patients and 10/16 (63%) of the multiphasic non-MS patients. This included children to require academic accommodations, for instance extra assistance at school, extra time to complete examinations and change to special education.

Of those who reported negatively affected school performance, 19/37 (51%) of the monophasic patients had CI assessed through an NPA, 7/16 (44%) of the MS patients and 6/10 (60%) of the multiphasic non-MS patients.

Furthermore, a total of 122/243 patients reported attention deficits in the standardized questionnaire.

#### Disease progression in MS patients

Three of the MS patients had an EDSS of 5.5 or above at the last moment of follow-up. All three patients received acute treatment at presentation. They all presented with a polyfocal CIS at the first event, including brainstem as well as spinal involvement (*n* = 3). These patients had a follow-up time of 24, 52 and 96 months and reached EDSS 5.5 at 6, 48 and 66 months, respectively. The first patient declined DMT. DMT was commenced in the other two patients, and both were escalated to second-line treatment (natalizumab) because of high MS disease activity.

## Discussion

We showed that the incidence of ADS and MS is higher in the period of 2011–2016 than of 2007–2010 [17]. Thirty-seven percent of the patients received a diagnosis of MS during follow-up, which is in line with previous reports about the proportion of MS diagnosis in ADS. Residual deficits are often reported not only in MS, but in all ADS subtypes at last follow-up, irrespective of the presenting phenotype.

The improved awareness of ADS in The Netherlands, aided by a more stable and extended referral network, likely attributed to the increase in incidence compared to 2007–2010. Notably, the small increase in ADS incidence was mainly driven by the rise in MS incidence. Our extended follow-up may have contributed to this higher MS incidence. We cannot exclude that the true incidence of paediatric MS is increasing in the Netherlands, as has been reported on overall MS incidence in other regions [18, 19]. Prolonged assessment of the incidence will be necessary to answer this question. Our new ADS incidence estimates are comparable to previous prospective studies that reported ADS and MS incidence in children [1, 24]. Moreover, our study confirms the skewed ethnic distribution in paediatric MS patients towards non-Caucasian ethnicities [1, 17, 25, 26].

A non-MS multiphasic disease course was observed in a minority of the patients (17/243, 7%). Remarkably 78% of these patients were tested seropositive for MOG-ab, in line with previous findings that MOG-ab positivity pleads against MS diagnosis and that these patients tend to have a relapsing disease course [27, 28].

MOG-ab and AQP4-ab seropositivity may be underestimated in this cohort, as the CBAs for both antibodies were developed and validated after the start of our prospective study. Serum was not retrospectively available of every patient who was included before the CBAs were implemented in routine diagnostics.

Over a median follow-up time of 61 months, only three MS patients reached an EDSS of 5.5 or above. However, residual neurological deficits are common in patients with MS (83%), in line with previous studies [11–16]. Cognitive deficits are commonly encountered in MS, but are also described in ADEM [29, 30]. Our results show similar results, as 34% of the ADEM patients show CI assessed by an NPA. A limitation here is that only one-third of our patients underwent an NPA in a standardized way. As part of the nationwide epidemiological orientation of our study, testing all patients was not feasible. Still, every patient who underwent an NPA had at least six cognitive domains tested. Furthermore, since 2013, all ADEM and MS patients who were presented in the paediatric MS centre in Rotterdam, have been consecutively referred for an NPA. Therefore, any selection bias within these two groups would have been minimal, leading to a more representative view on cognitive impairment in these patients. Also the multiphasic non-MS patients reported cognitive impairment and visual problems. These findings can be explained by the relatively high proportion of ADEM-ON patients in this group [6].

Our data further feed the impression that one single hit of ADEM can leave considerable intracerebral damage and may be considered less benign than previously thought [31–34]. A recent study showed reduced age-expected brain growth in monophasic ADS patients, especially ADEM, indicating irreversible and continuing changes occurring in the CNS even in the absence of chronicity [35]. Furthermore, recent studies have shown long-term residual deficits in ADEM and monophasic patients, such as a higher prevalence of motor problems, lower physical activity and fatigue [14, 15].

The high number of children with long-term residual deficits in the total group is concerning in relationship with school performances and psychomotor development, especially taking into account the cumulative nature of acquired disabilities after a longer disease duration in chronic demyelinating syndromes [9, 36]. Future participation in society, including work-related activities, is likely to be affected. Indeed, in a large proportion of adult patients, MS had negatively affected their employment situation [36, 37]. These effects could even be worse in paediatric onset ADS. Therefore, adequate detection and guidance of ADS patients is important to preserve and improve societal functioning, and is essential during follow-up of these patients into adulthood.

There are some limitations to this study. Despite our quite unique and extensive national paediatric MS network with full geographical coverage, it is still possible that we have missed a few cases and thus have an underestimation of our incidence figures. Adolescents with CIS may have been assessed and followed up by an adult neurologist and, therefore, have not been referred to take part in our study.

In addition, the negatively affected school performance in ADS patients may be correlated with other problems than CI. Fatigue, mood disorders, anxiety and negative coping strategies could correlate with a negative school performance in these patients, and may interact with cognitive impairment as well [14–16].

In conclusion, the reported incidence of ADS and MS in the Netherlands has increased during the previous years. Across all ADS subtypes the observed residual neurological deficits are considerable. Long-term follow-up studies of ADS patients will be needed to provide more insight into the risks involved and to identify possibilities for timely intervention.
